# What Motivates Speculators to Speculate?

**DOI:** 10.3390/e22010059

**Published:** 2019-12-31

**Authors:** Bedane S. Gemeda, Birhanu G. Abebe, Andrzej Paczoski, Yi Xie, Giuseppe T. Cirella

**Affiliations:** 1 Ethiopian Institute of Architecture, Building Construction and City Development, Addis Ababa University, 120 Addis Ababa, Ethiopia; bedanes@yahoo.com (B.S.G.); mesi.bire@gmail.com (B.G.A.); 2 Faculty of Economics, University of Gdansk, 81-824 Sopot, Poland; andrzej.paczoski@ug.edu.pl; 3 School of Economics and Management, Beijing Forestry University, No. 35, Qinghua East Road, Haidian District, Beijing 100083, China; yixie@bjfu.edu.cn

**Keywords:** speculation, land acquisition, motivation, real estate, development, Ethiopia

## Abstract

Land speculation that occurs on the urban border can be very problematic to the healthy development of cities—critical to economic growth. Speculative land investors, concerned with profits from trading in landed property, can especially affect developing countries where regulation is often poorly controlled and overly bureaucratic. An investigation into the factors motivating land speculators operating in the urban fringe of the city of Shashemene, Ethiopia is examined. The paper, in addition to contributing to the literature, is the second-known attempt and extension of the authors’ pilot research to study the behavior of land speculators in the urban fringe of a growing Ethiopian city. A theoretical framework and conceptual breakdown are put together with historical reference to early land speculation examples. Two questionnaires were separately administered with a representative random sample of 159 members from the local land developer association (i.e., investors) and 24 senior officials from the study area. A principal component analysis categorized the most significant dynamics in controlling land speculation procurements. Results indicated motivational reasoning as the prime cause for speculative activities. Evidence indicated that land speculation is a critical dynamic for self-worth especially with business-oriented persons. Entropy, the disorder of the communicative data, suggests a possible rethinking of the way government should intervene in the urban property market. As such, developmental smart cities in Ethiopia must thoroughly consider the dynamisms of speculative activities and its effects on local housing as it moves forward–in the 2020s.

## 1. Introduction

Land speculation is regarded as a critical issue in Ethiopia; thus, it is most welcome to enact the 1993 Land Use Act to address the land speculation problem and curb the incidence of growing land prices that are largely due to speculator activity. Nonetheless, this issue is far from being completely resolved as the issue of major acquisitions and land scarcity is still prevalent in many of the country’s urban and urban fringe areas [1,2]. Land management is essential in retrospect of city planning, land use development, and land speculation to create a successful cyclic system. Speculative land investors are those concerned with income from landed property trading, rather than with its use or earning capacity after growth, particularly in regard to housing which appears to be the highest and best use of the urban border [3,4,5]. This clearly implies that almost any investment aimed at a rapid accumulation of assets may be regarded as speculative [6]. Non-speculative investors, on the other hand, are those concerned with deriving profits from either property development or cash flow earnings received as a long-term investment from holding land [7,8]. Siegel [7] has stated that speculative investment can lead to an overvaluation of assets (i.e., securities or real estate) within a given area, which can often lead to an effect of property bubble-and-burst. Mohamed [9] described speculative activities as a form of fulfillment, a set of sub-optimal targets for which the desire of investors among developers has been widely observed. This occurrence tends to become a continuum as the policy responses needed to turn aside or alleviate a tightened situation are not as readily effective as anticipated. The main outcome of satisfying manners, as argued by Mohamed [9], are that many developers prefer projects located on green field sites (i.e., amenity-based or agricultural land) that take a relatively short time to dispose of in order to recoup the invested capital at a practical level of profit. This type of economic resolve can be more readily seen as urban building encroaches outwardly onto the urban border [10,11].

According to Gemeda, et al. [12], Shashemene’s much-acclaimed deficit of over 1000 housing units is projected to intensify the need for urgent land acquisition and growth to meet the current deficit. Nevertheless, some researchers (e.g., Golland and Boelhouwer [13] and Wang and Hua [14]) proclaim that developers’ land acquisition is not a specific response to demand; rather, developers take advantage of the general level of housing market activity and, in particular, “data signals” [13]. The State’s position in controlling land acquisition appears to affect the motivation of acquisitions by speculators, either due to the fact that land can provide a source of profit through land banking or land trading or as a development factor. Recently, from observation, investment activities tend to have increased in frequency due to rising urban populations and anticipation and hype surrounding urban fringe areas (i.e., mostly as a result of new road infrastructure). Other drivers of speculative activities include close proximity of bare land to an urban center, excitement and hype surrounding the location’s commercial viability, overestimation of land demand, and the notion that the location is a viable growth investment area within the development plan of the city. Eventually, speculators may buy the land and use it immediately for a completely different purpose, which defies logic in order to lay claim to it, such as establishing a mechanical factory, operating a vegetable farm, erecting a perimeter fence, or building on-site quarters for servants (e.g., security guards). These are some provisional activities frequently carried out by land speculators after valid purchase in order to “legitimize” their proprietorship of the land in line with Ethiopia’s jurisdictional definition of development.

Dealings in the land market can be an indication of the idiosyncrasy of the environment as well as the area where it is situated. Two market types exist as both central and peri-urban [15,16]—classified as sale and rental markets. According to the World Bank [16], a sales market is when freehold interest can be sold while a rental market refers to when “usage rights” are temporarily reassigned for a limited period. While land speculators are inclined towards the sales market for transferability of freehold interest, it should be noted that many societies also allow for both partial and total disposal of property leasehold interest. This study, in an umbrella-like effect, oversees such dynamics motivating land speculators operating in Shashemene’s urban fringe. As such, Ethiopia’s Proclamation No. 721/2011 of its Land Use Act allows a lessee to assign their unexpired interest in land with consent from the State; thus, allowing the market to be either formal and structured or informal and operating extra-legally [17,18,19]. A formal land market follows a set structured rules and regulations while participation in the market abides by legal procedures both for acquisition and disposal. On the other hand, the informal market often outweighs land nationalization since it is not always fixated with strict pertinent rules commonly found in many developing countries where property rights are often constricted [20,21,22].

In the city of Shashemene, the legal occupier of any land will not only have lawful title to it (i.e., a freehold, leasehold, or license from the actual landowner), but also need formal planning approval for any buildings or other erected structures permanently affixed to the land, including land use of which those structures pertain to. As defined by the Business Dictionary [23], development is “the carrying out of the building, engineering, mining, or other operations in, on, over, or under land, or the making of any material change in the use of any buildings or other land.” According to the statutory definition, building operations refer to the demolition of buildings, rebuilding, structural alterations of, or additions to buildings and other operations such as “excavation or other works carried out on the land, including fencing” [17]. Thus, in accordance with Ethiopia’s Land Use Act, it is important to note that, since a land speculator can effectively prevent their land from being repossessed by the original seller (i.e., for failing to develop within two years), they “employ somewhat deceptive-like activity” [17]. Experience indicates that unprincipled speculators can ensue and process a genuine (i.e., legal) land title and acquire planning permission for a structure or improvement (i.e., even an ordinary fence) all in an attempt to retain legal ownership and procure capital appreciation.

The motivation of the research is focused on property market dynamism. It increasingly has become intertwined with speculative financial flows and has shaped (and reshaped) urban land under the stimulus of capital over accumulation with the intention of absorbing surplus (i.e., a process that ironically can be a source of economic macro crises as well as a share of the property market as gross domestic product increases). In this context, Ethiopia is certainly not excluded from urban development and speculation despite its ever-increasing level of urbanization—especially since the 1960s. In light of this, land speculators are harvesting huge revenue from urban and peri-urban areas by keeping land vacant. Land speculation in this regard is seen as a critical issue in Ethiopia; hence, it is appropriate to enact the Land Use Act to address the problem and curb the incidence of rising land prices that by in large spike due to speculative activity. However, this issue is far from being fully resolved as the problem of large acquisitions and land deprivation is still endemic in throughout the country’s urban fringes [12]. To resolve this problem and increase the benefit to society, this study aimed at looking into the motivational factors behind land speculators using Ethiopia as a case study. We look at the motives that motivate land speculators in the urban fringe with the position of making policy recommendations in line with speculation-based best practices. A breakdown of the paper is structured as follows: Section 2 explores the theoretical frame and conceptual clarification of land speculation, Section 3 contains the methodology, Section 4 elucidates the results and discussion, and Section 5 provides the conclusions.

## 2. Theoretical Frame and Conceptual Clarification

**Figure 1:**
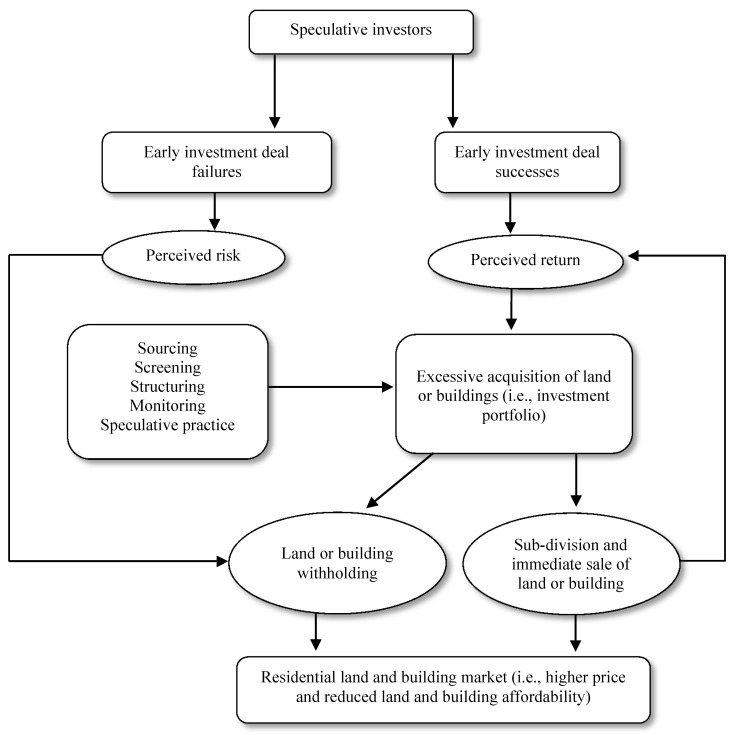
A cognitive model of speculative investors’ operations in property sectors, adapted from Valliere and Peterson [31].

The theoretical frame is provided by insights gleaned from the classical concentric zone model [24], sector or axial development model [25], and Alonso’s [26] bid rent model—all which seek to clarify why detailed land use is situated where they are in the cityscape. Burgess’ [24,27] model portrays residential land use structures by examining how one might plan the layout of a city [28]; out-of-date, it overlooks the importance of transport routes, site, and physical uniqueness in shaping a city’s evolution and urban border zones. In terms of the concentric model, weaknesses are remedied by land use zones that focalize on key urban areas associated along central arterial transportation lines. From this theoretical aspect, and as already identified in the analysis part by principal component analysis, land speculators are motivated by regulatory lapses, location preference, informality of land title, and inexpensive land. Those variables vary depending on the model. For instance, within the context of transportation routes, where infrastructure is usually found, speculators are motivated to hoard more land. Moreover, as described by Hoyt [25,29] in which the major land use zones in the urban area is “aligned along the major arterial transportation routes” [29], land speculators in parallel follow infrastructure (i.e., transportation, roads, etc.) when modeling city advancement. Likewise, with axial theory, current major transport facilities also control urban development via two factors: speed and pattern. This has meant that, while an area may be remote, once it has direct access to major roads (i.e., in terms of time taken to and from the central business district), a transition zone is likely to materialize which, potentially, can act as a target for land speculators. In practice, however, the impacts of externalities such as rising urban population, rising demand for land, and the desire for business profit can oblige land speculators to consider holding the land for a block of time in order to ensure its appreciation—that is, introducing the notion of “mastering the silent game” [30] of a risk-return trade-off in order to break-even (Figure 1).

**Figure 2:**
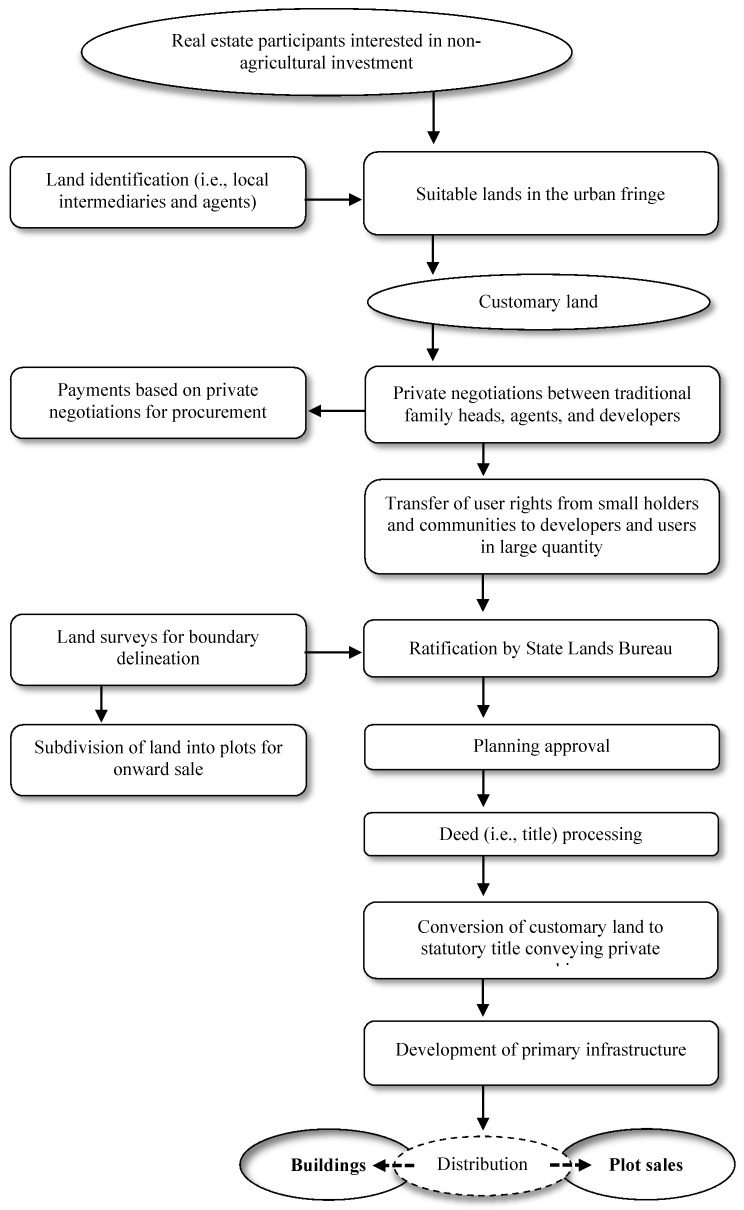
Land acquisition and legalization procedures in Shashemene.

Conceptually, land speculation may be defined as a function of the ineptitude of land policies and the low believability of land transactions taking place in an urban tassel. For instance, land policies and their efficacy rely on urban and regional planning procedures and interrelated policies and strategies that regulate and control the economic and social practices on property and land assignment. This is achieved through proper enforcement of “building regulations, planning standards, and the zoning bylaw guiding [specific land uses and] infrastructure provision” [32], the lack of which may result in confounded and poor development patterns along the urban border. Accordingly, best practices would make certain that planning tools are judiciously practical as both precautionary and counteractive measures for ensuring proper and effective land use deter sub-standard, natural, and chaotic development and pointless delays in project development completion. As a result, the physical, spatio-temporal and social uniqueness of a community are vital criteria for thoughtfulness in reforming the urban border [32]. A follow-up notion, within this framing, is land transactions (i.e., mortgage transactions that use property as collateral for bank loans). Specifically, land transactions experiencing changes regarding their arbitrative status, beginning from “an intention to acquire and proceed to the procurement and certification of land title” [33]. Urban fringe land is often perceived as marginal land, available but “uncultivated” [34] and “suitable for investments” [35] other than agriculture [36,37]. As a result, its availability in an uncultivated, large-scale form—outside the scope of agriculture—prompted the conceptual framing of Figure 2. As such, the real cause of speculation is the specified expectation of the augmentation of land value, which occurs in all advancing countries from the stable increase of rent, which leads to speculation, or the “holding of land for a higher price than it would then otherwise bring” [38]. In contrast, the consequences of land speculation are “tenantry and debt to farms, and slums to luxury in cities” [39].

Large-scale land hoarding, throughout the developing world, stresses competition for land and results in land disputes due to escalating food prices and the necessity to cultivate more land for agriculture, “uncontrolled rural-urban immigration leading to higher population densities” [40], and increasing demand for shelter and other forms of accommodation [3,33,40,41]. According to Colin [42], numerous large land acquisitions along the urban border involve prolonged negotiation processes which revolve around hoarding of long-term rights of ownership. In Ethiopia, long leaseholds of 99 years or other agreeable titles are only decided by the State, in observance with the provisions of the Land Use Act No. 4 of 1993. The Act, as observed by Hurni, et al. [43], was promulgated following a report submitted to the Federal Government by which a land use panel examined the current system of land occupancy and advocated measures for curbing the activities of land speculators, “by making it easier for the government to acquire land” [43] for development projects nationwide, including the capital. Typical large land acquisition involves identification, negotiation, procurement, title, and distribution or subdivision. Suitable privately-owned land is first recognized through consultation with local intermediaries (i.e., traditional authorities), in which mostly local agents act as intercessors. In Shashemene’s urban fringe, some developers with the support of local go-betweens (i.e., partners) instigate direct communication with local traditional chiefs (i.e., including heads of families) when they wish to acquire land which usually instigated via a back-and-forth. It should be noted that many land deals in Ethiopia’s urban tassel are classified as “semi-formal and customary” [42] rather than formal tenure arrangements—prevalent in many African countries [44,45].

After settling the contracted purchase agreement, the owner is directed to submit it to the Lands Bureau for registration of title. Additional documents that must be succumbed including a survey plan indicating the beacons and boundaries of the land, copy of the approved building plan, and, if necessary, an environmental impact assessment report on the proposed project for the site. Once the submission process is ratified, land and building certification can be issued. Recently published research by Gemeda, et al. [12] is a first attempt to piece together some of the roles land speculators pose within the country. This paper acts as an expansion to those findings by furthering the research and nature of land speculation in Ethiopia’s major cities (i.e., in respect to urban and urban fringe areas). A core focus is to make policy recommendations in the interest of city livability and sustainability. 

**Figure 3:**
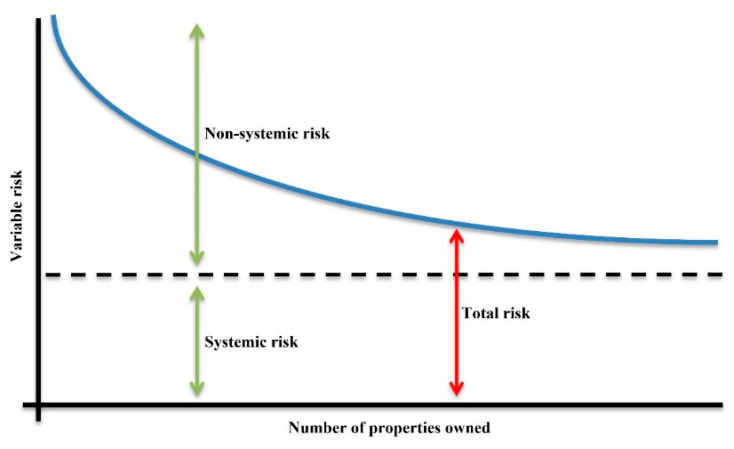
Variable risk versus number of properties owned as a measure of non-systemic and systemic risk (i.e., total risk).

Speculators, according to Andreasson, et al. [46], are likened to “high-risk traders almost of the same kind to gamblers” [46], whereas lower risk investments grounded on core research and analysis fall into the category of desirable investments. Subject to an in-depth analysis, a good investment should “swear the safety of the capital invested in addition to ample income and yield return” [46]. On the other hand, investment processes not up to scratch with these requirements are termed as speculative in nature. In terms of systemic risk, the possibility of an event triggering severe instability or collapse of an economy, in retrospect, plays with the speculative idea of “too big to fail”. Often systemic risk can be used as a justification for government to intervene in the economy. The basis for this intervention is the belief that government can reduce or minimize the ripple effect during turbulent economic times [47,48]. As such, an important framing of variable risk in relation the number of properties owned (i.e., held) is correlative to total risk which starts off as non-systemic risk and augments to systemic risk as owned properties numbers increase (Figure 3). Sometimes, however, it has been found that non-intervention in the economy can be beneficial solely based upon the fact that if variable risk is too volatile, not doing anything (i.e., leaving the land market to sort itself) is best. This is more often the exception than the rule since it can destabilize the land market, more than projected, due to speculator sentiment [47,48,49,50].

Investor characteristics differ from those of a speculator, in so that, an investor “acquires land as a factor of production” [51], whereas a speculator attains land in the hope of profiting from an upsurge in its market value (i.e., capital appreciation at the end of a holding period). Speculation is not caused by a shift in demand due to change in taste, fashion, consumer need, or supply. As argued by Knittel and Pindyck [51], a shift in fundamentals can frequently trigger a transformation in price, an outcome not necessarily a determinant of land market speculation. As such, speculative demand and pressure can force changes in the property market due unavoidable external influences that simultaneously structure urban fringe areas regardless of government policy.

Participants involved in speculative practices are grouped into three classified levels by Triantafyllopoulos [52]. First, there are those who are “informed speculators” [52] in the sense that they have access to both public and private information. They are otherwise known as public officials in charge of land allocation (i.e., plots and buildings). Second, there are those who are “un-informed speculators” [52]—these are persons who only have access to public information and are otherwise known as either investors or developers. Third, there are “private purchasers” [52] who are individuals that are not information-driven. In practice, the different functions played by each of the actors can particularly alter the cityscape and overall layout of how cities interact. For example, developers may be regarded as investors who initiate and carry out land development projects which can play an active and leading role in the development of the urban fringe, perceived as the prime “sculptors of spatial structures” [53] of any open space. Jonas and Wilson [54], Logan and Molotch [55], and Molotch [6] refer to these developers as “growth machines” and directly link speculative practices with the conditions for how cities grow.

Generally, land speculation depends on endogenous and exogenous factors. Endogenous factors can be related to the institutional framework of any given country (i.e., property rights protect by law, enterprises dealing in land markets, and types of land markets—e.g., regulated or free). Exogenous factors are much more complex. They reflect the international position of any given country, situation, and perspective of its economy, political stabilization, social and human capital (i.e., its approach and control of nationally-owned land), and foreign investment [56,57,58]. These influences forecast concerns and potential land and building prices as well as project an overall perception on the type of land speculation at the city-level. As such, popular and scholarly housing debates, for example in London, are concentrated on the super-rich as stated by Atkinson [59], Hay [60], and Hay and Muller [61] in terms of patterns of consumption, economic power, and political control [62,63]. Moreover, parks are considered safe havens for park property [64] and produce rental income that often outgrows inflation compared to lower interest rates on savings accounts and weaker returns on investment of financial products [65]. There is evidence that, since the early 1990s, middle-class buyers from Hong Kong have been investing in London’s main property market as noted by Ho and Atkinson [65]. Although Hong Kong’s first wave of investors primarily focused on property for their own use, second and third wave investors emerging between 2000 and 2009 were interested in financial returns (i.e., short-term investment or long-term rentals). The scale of this investment activity has become somewhat significant since Hong Kong shareholders, by 2012, have already purchased about one in six new residential properties sold in central London [66]. Significantly, an estimated 514,000 Hong Kongers invested in property outside Hong Kong in 2016 [67], representing 7% of Hong Kong’s population at around 7.4 million [68], a trending figure that continues to increase. Similar issues (e.g., in regards to housing affordability in Sydney, Australia [69,70,71] and Vancouver, Canada [56,72,73]) parallel the global crisis of urban land speculation practices in which “accelerated (re)urbanization of capital and people [has led to] the provision of cheap credit and the rise of intra-society inequality” [56]. Nonetheless, global real estate is considered a class of capital for which investors “diversify their investment portfolios” [74] often at the expense of local community housing affordability interrelated via political and economic spheres.

## 3. Methodology

**Figure 4:**
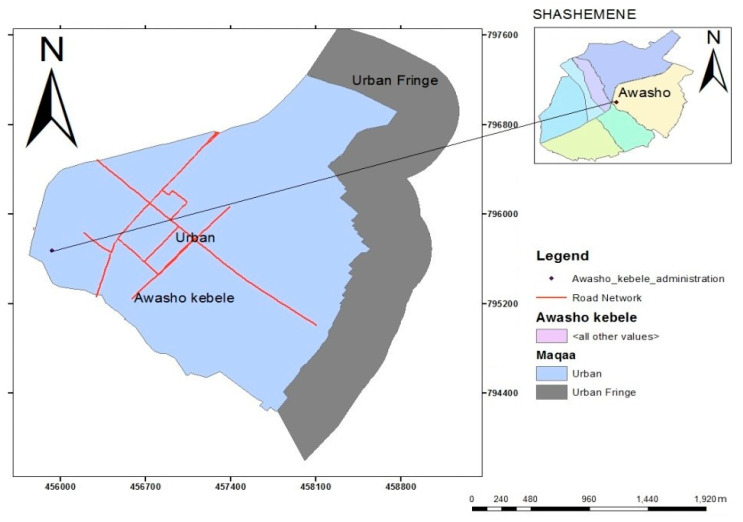
Map of the study area.

There are six main urban fringes in the greater city area of Shashemene (Figure 4). Of these areas, a number of peri-urban villages (i.e., Awasho, Arada, Abosto, and Bulchana) exist. These peri-urban areas stretch adjacently along the Awasho asphalt road in the east, Bulchana in the southeast, and Alelu, Dida-Boke, and Arada in the neighboring areas of the northwest. The Awasho urban fringe in the southeast was selected as the study site due to its fast-growing hub-like industrial and residential activities. Awasho is also a major transportation route currently coping with the impact of rapid urbanization where investors and foreign industrialists are favoring to locate. Pressure and demand for residential land, even though it is unaffordable due to unnecessary land withholding and price increase (i.e., created by land speculators), is seen to be physically available.

Primary data were collected through a combination of methods including self-administered questionnaires, direct observations, and in-depth interviews, using two types of questionnaires. The first set of questionnaires was randomly given out to the local land developer association (i.e., investors) in which the sampling frame was 159 investors—confirming the sample size determination was free of bias or error of misclassification [75,76]. The structure focused on the motivational dynamics for large-scale land purchases, reasons for their holdings (i.e., after procurement), period of time between procurement and actual development (i.e., if any), land status (i.e., procured by developers), legal title, and period between procurement and further subdivision (i.e., for onward sale without further development).

The second set of questionnaires was directed at 24 senior officials of the Shashemene City Land Agency who reported upon whether any effective policies were put in place for limiting (i.e., controlling) developer activities with a tendency towards speculative activity. Further inquiry looked at what approaches and policies had been used as well as their success rate. The samples represented the target population and not the total population. To regulate the average mean of the developers, we employed a five-point Likert-type rating scale that considered cause and control of land speculation city-wide. Reliability of the questionnaire included a Guttman Split-half test to control for internal consistency. To end, principal components analysis (PCA) was exercised on vital factors germane to large land procurements. Specifically, the PCA method used orthogonal transformation to convert the set of land acquisition variables into a set of values of principal components.

## 4. Results and Discussion

### 4.1. Factors Motivating Land Speculators 

Essentially, land speculation is holding land primarily to meet future demand and not present need. The aim of the speculator is to create a synthetic value that in itself is unacceptable. Recent activities leading to large-scale land acquisitions and withholding in many urban fringes have triggered price rises in the market, intensified the inelasticity of supply of developable land, and complicated the already disorganized housing issue (i.e., affordability for low-income earners). The provision of affordable housing cannot be left exclusively to private developers as government has a major role in ensuring equity and effectiveness in the allocation of land resources by way of externalities [75]. In analyzing the motive behind speculative activities and land use control in the urban and urban fringe of Shashemene, the following dimensions were discussed as a means of contributing to knowledge: prominent characteristics of land acquisition, range of time between actual land acquisition and development by speculators, and factors responsible for speculative land acquisition.

### 4.2. Prominent Characteristics of Land Acquisition

Respondents to the land developer questionnaire were predominately private developers (i.e., 85%). The status of land bought by developers on the average shown to be left vacant and underutilized (i.e., undeveloped) was more than seventeen years. Moreover, only four of the lands bought have adequate legal title in the form of the governor’s consent. Others either have an illegal receipt of purchase or have been registered in the State gazette as evidence of government approval, while others do not even have any certification. Some of the developers indicated that they usually subdivide their land immediately after purchase (i.e., 25.2%), while 65.6% subdivide within a two-year period. This suggests most of the respondents did not have an aim in commencing concrete development at the time of purchasing land. The location of acquired land is spread over the whole of the study area with respect to individual developers’ needs and requirements, usually dependent on accessibility and proximity to infrastructural services.

The oldest land was 400 m^2^ purchased in 1991 that has remained vacant for 29 years since its purchase. The most recent and largest purchase of 6900 m^2^ was done in 2015 with government consent, but has remained vacant ever since. The cheapest acquisition was secured from a family by private treaty (i.e., negotiation) for 875 Ethiopian Birr (i.e., US$ 30.13) per m^2^, while the costliest acquisitions closed at 5000 Ethiopian Birr (i.e., US$ 172.18), backed by government consent. Only four of the acquired lands are either fully or partially developed for housing (Table 1). As such, it can be inferred that developers (i.e., local speculators) hoarding land and taking it off market for long periods of time also have a relational connection to housing rental income and the overall housing market. As it is commonly portrayed in urban economics, the demand for land is driven by the demand for housing [56,63]. As the price of land increases, the price of housing follows and vice versa, which was commonly seen in Ethiopia’s land market. Moreover, land markets would thus have a positive impact on improving land access by land-poor households. As long as imperfections affect only one market, all relevant actors still have the opportunity to cultivate the same amount of land per capita. However, credit market imperfections can offset or even eliminate supervision cost advantages of family farmers.

### 4.3. Range of Time between Actual Land Acquisition and Development by Speculators

In reference to Table 1, the range of time between actual land acquisition and development by speculators shows a revealing reality that the four developed properties bought in 1995, 1997, 1998, and 2000 were developed a whopping 19, 9, 19, and 15 years later, respectively. These numbers correlate with the average, to date, mean status of land bought by developers that has been left vacant and underutilized (i.e., seventeen years). This confirms our earlier misgiving that most of the land speculators in the study area do not seriously intend on undertaking development within the short-term following their acquisition. This ability to hold on to land for long periods of time points at the low risk presented suggests that land speculators tend to delay development in the expectation that a buyer will show up eventually. As such, it has been noted that, when speculators’ hope of finding a buyer is reduced, they resort to the legitimization of their ownership by spurious or unscrupulous land enhancements, including the erection of just an ordinary perimeter fence.

### 4.4. Factors Responsible for Speculative Land Acquisition

Speculators were asked to indicate the factors motivating large land acquisition in which more than 20 reasons were ranked using a Likert scale. Responses obtained were analyzed using PCA to signal reasons for significant factors relevant to large land acquisitions. As such, seven principal components were identified during this process (Table 2). As a widely used statistical technique, PCA is useful for determining the latent variables of the obvious variables [75]. The results of the principal components are sensitive to the relative scaling of the original variables.

**Figure 5:**
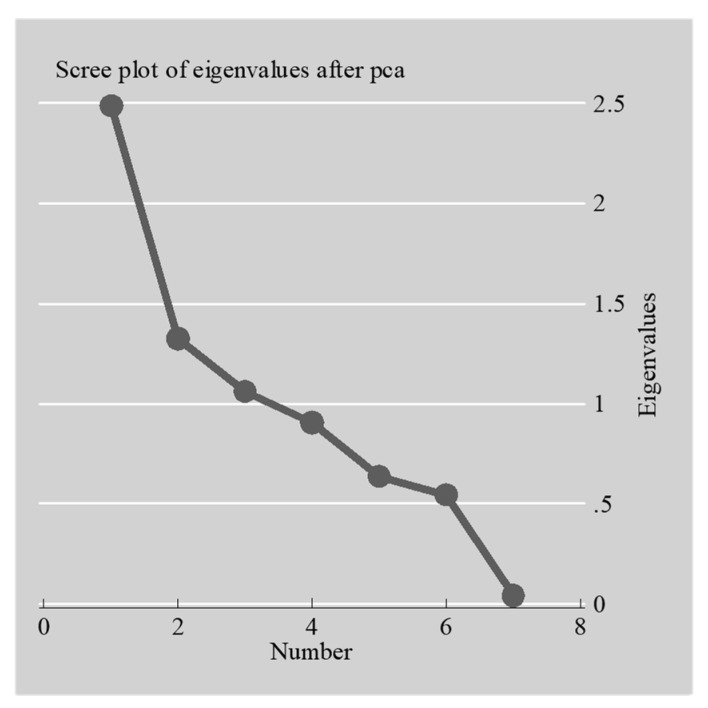
Scree plot of land speculation in the Shashemene urban fringe.

From the PCA, two types of findings can be noted. First, anti-image diagonals illustrated sampling adequacy via the Kaiser–Meyer–Olkin measure (i.e., with a value of 0.60 for each variable), while Bartlett’s test of sphericity indicated a significance *p*-value of 0.000—both demonstrating a significant level of sample tolerability for this analysis (Table 3). Second, a total of seven principal components were recognized with a collective variance of 69.59% in which specific high impact findings originate from the first, second, and third components (Table 4). Out of the three factors studied, the first two together explained 54.42% of variation compared to 69.59 % explained by unrotated factors, as indicated in the Scree plot in Figure 5.

Correlating the Scree plot of land with Table 2 shows the rotated component factors. The rotated component matrix specifies sufficient loading on all seven components. There are two dominant variables on the first component (i.e., PCA 1) due to speculators who bought land in close proximity to a developed area as well as regulatory lapses make it possible to buy land cheaply. The dominant variables on the second component (i.e., PCA 2) were good profiteering business efforts and the high possibility of rising prices for profit maximization. In the third component (i.e., PCA 3), the dominant variable was spectators’ ability to buy land due to ease of access of obtaining a land title. Next, privilege information on zoning and planning of a new development scheme best denoted the fourth component (i.e., PCA 4). Good investment for buying land and being able to keep it (i.e., as long as the value keeps increasing) before selling it as well as privilege information on zoning and planning of a new development scheme were the dominant variables in PCA 5 and PCA 6, respectively. Lastly, regulatory lapses making it possible to buy land cheaply was dominant in PCA 7. The common variables that constantly occur among the components were: (1) privilege information on zoning and planning of a new development scheme and (2) regulatory lapses that made it possible to buy land cheaply. However, it is unusual that, of all the rated factors, 88% of the responses did not feel that the demand was being met by government allocation. In other words, the usual acclaimed motivational reason for land acquisition of excess demand not met by government allocation rated very low (i.e., low probability) and did not even feature more than once (i.e., except in PCA 4). This entropy, disorder of communicative data, suggests disparity between the hidden intention of speculators [77,78] and what really exists physically on the ground. According to Zhang [79], surprisal information on “price histories can be used to predict near future returns with a probability better than [that of] random chance”. As such, since market participants are separated between producers and speculators—the former postulates “negative entropy into the price, upon which the latter feed” [79]. Residual negative entropy suggests an urgent need for more effective government intervention in allocating land in the urban land market, particularly due to land scarcity and relating price mechanism ill-equipped in maintaining a standard. 

In reference to Mercer’s [80] definition of livability (i.e., “a concept that assesses which locations around the world provide the best or the worst living conditions” [80]), livability combines a range of benefits that include benchmarking perceptions of development to assigning hardship allowance as part of expatriate relocation packages. Critics of this view have argued that “no city in the world is really excellent and that livability is only a relative term” [80]. In the context of this paper, it is contended that land speculation is detrimental to a city’s livability (i.e., sustainability) in several ways as proclaimed by Swierenga’s [81] research on land speculation and impact on American economic growth and welfare. He states that land speculation gives rise to green pockets that are easily transformed into high crime areas fostering slum development by way of rural-urban migration and accentuating a lack of housing as a result of holding developable land. At length, Swierenga’s [81] findings parallel, to some degree, Shashemene’s reduced property tax base since a number of American municipality’s often do not tax vacant land or are not subject to property tax (i.e., they are subject to a tenement rate), which can result in a loss of potential sources of revenue. 

## 5. Conclusions

We conclude that, for the city of Shashemene, the government is fully aware of speculative practices occurring along its border areas. Government policy put in place for regulating excessive land acquisition is still limited to the Land Use Act of 1993. It is not clear how this legislation will deal with restricting or ridding speculative activities throughout the city’s limits. As of now, over the past few decades, legislative implementation or political clout has failed to decrease this phenomenon. Government has not effectively controlled or penalized excessive land acquisitions that are not for immediate use. Our results verify a relatively low level of property rights formalization, mainly due to bureaucratic practices and corruption that encourage large acquisitions of land with impunity. This problem demonstrates a disturbing trend in the dynamic of the city’s fringe areas mostly due to repeated failures of government in regulating land use activities. As a result, there is an urgent need for more effective government intervention in allocating land in the urban land market, particularly due to land scarcity and relating price mechanism ill-equipped in maintaining a standard. In addition, there is the need for strategic measures, such as the institutionalization of public–private joint ventures that will not only work in partnership with landowners and developers, but also work to develop a “speedy, efficient, and sustainable management” [46] and development of the fringes in terms of provision of cost-effective infrastructure and increased public information on land market opportunities in the fringe areas. Toward this end, the creation of a government holding division to procure vacant lands is believed to be a needed priority.

To minimize land speculation seen in urban and peri-urban areas, the following two points are recommended: (1) policy implementation of “one-man-on plot” rule, which implies that the number of plots given to residents must be restricted and (2) there should be imposition of a land value tax (LVT). These two points would significantly curb excessive land speculation and allow for controlled development to occur. As a result, a number of benefits on enforcing a high LVT, include: high annual overhead cost to the land speculator that would either lower present values or provide encouragement for quick disposal, especially if landowners could not meet the overhead costs and pessimistic future appreciation of land value. Moreover, Clawson [82] argues that sprawl supplemented by land speculation is fruitless in that it absorbs “capital, manpower, and entrepreneurial skills without proportionate public gains” [82]. Based on our findings, it is contended that the need for strict regular site inspections and a corruption-free regulatory regime are clearly critical in monitoring land speculative activities in Shashemene. Underlying these suggestions is the idea that the government, at some level, retains significant powers for influencing, if not controlling, the future form of the outer edges. To achieve positive goals, speculation on urban fringe lands must be significantly decreased or eliminated. The net effect of these various instruments would be to greatly alter the general probability (i.e., prospect) of land actors on speculation. Entropy theory suggests disparity between the hidden intention of speculators [52,53] in which the historicity of price can be used to predict near future earnings with a probability that is better than random chance [54]. As a result, the timely implementation of an LVT policy could lead to more predictable land prices, extemporaneous housing, or other land use advances. In this process, some of the private validations for land speculation would be overcome. Additionally, since the eventual purpose of speculation is short-term capital gains, one way to stop speculation could be through effective government’s association with land-based “pressure groups” [82] or non-profit organizations regularly monitoring the border areas located in the city’s domain. Furthermore, support for landowners to initiate direct development within a sensible period after the purchase could be a secondary approach. Finally, lending institutions (i.e., banks and insurance companies) should be vigorously involved in the push towards easing conditions placed on mortgage applicants.

## Figures and Tables

**Table 1:** The status of lands bought by speculative developers.

Land Bought (m^2^)	Location	Year Purchased	Year Developed	^†^ Price per m^2^	Document Title When Purchased	Status	Vacant Period (Years)
160	Awasho	1997	not developed	2000	illegal receipt	Vacant	23
220	Awasho	1998	not developed	1400	illegal receipt	Vacant	22
320	Awasho	2000	not developed	1000	illegal receipt	Vacant	19
350	Awasho	2001	not developed	2500	illegal receipt	Underutilized	18
200	Awasho	2004	not developed	4000	illegal receipt	Vacant	16
6900	Awasho	2015	not developed	5000	legal receipt	Vacant	5
350	Alelu	2001	not developed	4000	family receipt	Vacant	19
250	Alelu	2005	not developed	3500	illegal receipt	Vacant	15
140	Alelu	2013	not developed	2500	legal receipt	Vacant	7
500	Arada	1997	not developed	3500	illegal receipt	Vacant	23
450	Arada	1995	2013	4500	illegal receipt	developed	19
400	Bulchana	1991	not developed	875	family receipt	Underutilized	29
320	Bulchana	1998	2016	3500	illegal receipt	Developed	19
140	Dida Boke	1997	2005	4500	illegal receipt	Developed	9
250	Dida Boke	2000	2014	3500	illegal receipt	Developed	15
250	Dida Boke	2004	not developed	980	legal receipt	Vacant	16
200	Kuyera	2011	not developed	1050	legal receipt	Vacant	9

^†^ US$ 1.00 = 28.7 Ethiopian Birr; Source: field survey.

**Table 2:** Principal components matrix of reasons for large-scale land acquisitions ^‡^.

^†^ Variable	Comp1	Comp2	Comp 3	Comp 4	Comp 5	Comp 6	Comp 7	Unexplained
Q1	−0.2115	0.6684	−0.1562	0.1136	−0.1684	−0.6653	0.0144	0
Q2	−0.3811	0.0490	0.4494	−0.1927	0.7660	−0.1616	0.0190	0
Q3	−0.1903	0.1367	0.7892	0.2840	−0.4580	0.1774	0.0201	0
Q4	−0.2884	0.5696	−0.2687	0.0696	0.1431	0.7032	0.0192	0
Q5	0.2873	−0.0128	−0.0217	0.8822	0.3697	−0.0423	−0.0089	0
Q6	0.5470	0.3424	0.2116	−0.2120	0.0973	0.0461	−0.6984	0
Q7	0.5607	0.3004	0.1827	−0.2032	0.0918	0.0383	0.7147	0

^†^ Q1: good profiteering business and high possibility of rising price, Q2: good investment to buy land and keep it (i.e., as long as the value keeps increasing) before selling it, Q3: bought land due to ease of access of obtaining land title, Q4: privilege information on zoning and planning of a new development scheme, Q5: excess demand not met by government allocation, Q6: bought land due to its close proximity to a developed area, Q7: regulatory lapses makes it possible to buy land cheaply; ^‡^ data displayed factorability potential based on Bartlett’s test of sphericity, employing a chi-square value of 192.773 at 21 degrees of freedom, significant at 0.01 showing correlations among the chosen variables, hence a supportive criterion for factorability.

**Table 3:** Two calculations: Kaiser–Meyer–Olkin measure and Bartlett’s test.

Kaiser-Meyer-Olkin Measure of Sampling Adequacy		0.60
Bartlett’s test of sphericity	approx. chi-square	192.773
-	df	21
-	sig	0.000

**Table 4:** Total variance explained for the initial eigenvalues.

Component	Initial Eigenvalues		Cumulative Percentage of Variance
	Total	Percent of Variance	
1	2.48345	35.48	35.48
2	1.32565	18.94	54.42
3	1.0619	15.17	69.59
4	0.907426	12.96	82.55
5	0.636939	9.10	91.65
6	0.544361	7.78	99.42
7	0.0402671	0.58	100.00
